# Genome description of a potentially novel species of Rossellomorea sp. strain H39_3 isolated from the Hindon River, India

**DOI:** 10.1099/acmi.0.001042.v4

**Published:** 2026-05-19

**Authors:** Chakresh Kumar, Nirupama Saini, Anwesha Ghosh, Punyasloke Bhadury

**Affiliations:** 1 Integrative Taxonomy and Microbial Ecology Research Group, Department of Biological Sciences, Indian Institute of Science Education and Research Kolkata, Mohanpur-741246, Nadia, WB, India; 2 Centre for Climate and Environmental Studies, Indian Institute of Science Education and Research Kolkata, Mohanpur-741246, Nadia, WB, India

**Keywords:** arsenic, bioremediation, polysaccharide, river, *Rossellomorea*

## Abstract

In October 2024, a putative novel species, belonging to the genus *Rossellomorea*, designated as strain H39_3, was isolated using Luria–Bertani medium from surface water representing station H39, located on the Hindon River, in close proximity to Gautam Buddha Nagar in Uttar Pradesh, India. The 16S rRNA sequence of this isolate showed 100% identity to *Rossellomorea marisflavi* from the International Nucleotide Sequence Database Collaboration (INSDC) DNA databases (GenBank/DDBJ/ENA). Whole-genome sequencing was undertaken using long-read sequencing with Oxford Nanopore Technologies (ONT) chemistry on the MinION platform, followed by genome annotation against the NCBI Reference Sequence Database (RefSeq) and Genome Taxonomy Database (GTDB) databases. The genome is ~4.46 Mb in size, with a G+C content of 48.64 mol%. The low average nucleotide identity (92.23%) and digital DNA–DNA hybridization (46.1%) values showed affiliation with the reference strain * R. marisflavi,* indicating the isolate as a potential novel species. Functional analysis of the draft genome of this isolate revealed an array of genes, including the presence of *ars*C (arsenate reductase), the assimilatory nitrate reduction pathway and the ability to degrade polysaccharides. The presence of nitrogen metabolizing genes such as *nir*B (nitrite reductase subunit B), along with the ability to break down complex forms of carbon, offers the potential of this strain for application in bioremediation of contaminated river ecosystems.

## Data Availability

The whole-genome sequence was uploaded to National Center for Biotechnology Information (NCBI) under the accession no. SAMN47866444. The corresponding raw sequencing reads are available in Sequence Read Archive (SRA) under accession no. SRR34443858.

## Introduction

In the family Bacillaceae (Phylum: Firmicutes), the genus *Rossellomorea* was established in 2020 and includes species that were previously categorized as *Bacillus* [1]. It includes previously described *Bacillus* species that can be reliably identified from all other Bacillaceae species by the presence of exclusively shared conserved signature indels in their protein sequences [2]. With the genus *Rossellomorea*, half of the species have been isolated from diverse marine ecosystems and are heterotrophic, halophilic or halotolerant aerobes [2,4]. The genus *Rossellomorea* comprises Gram-positive, rod-shaped bacteria [5]. It shares a close relationship with the genus *Lactobacillaceae* of lactic acid-producing bacteria. To date, valid names representing six species within this genus have been reported. These include the type strain *Rossellomorea aquimaris* and *Rossellomorea marisflavi*, which were first isolated from a tidal flat [5]. Besides, *Rossellomorea arthrocnemi* has been discovered from plant roots [2], *Rossellomorea vietnamensis* from Vietnamese fish sauce [6], *Rossellomorea oryzaecorticis* from rice husks [7] and *Rossellomorea pakistanensis* from a salt mine [8]. Given the versatile distribution of this genus, there are opportunities to further isolate members of this genus from contaminated freshwater ecosystems and investigate their bioremediation potential for restoration of these ecosystems.

## Methods

### Sampling and isolation

Surface water sample was collected from the Hindon River, near Gautam Buddha Nagar, Ghaziabad, Uttar Pradesh, India (28° 24' 52.6" N; 077° 28' 53.0" E), during the month of October 2024. The collection was done in a 50 ml sterilized centrifuge tube (Tarsons, India). The Hindon River is reeling from anthropogenic forcings such as continuous flow of untreated municipal sewage and industrial effluents. The *in situ* environmental parameters, such as surface water temperature (SWT), pH, dissolved oxygen, total dissolved solids, electrical conductivity, water depth and water velocity, were also measured at the time of sampling. SWT was measured using a digital thermometer (Digi-sense RTD Meter 20250–95, Cole-Parmer, United States of America), and pH was recorded using an EcoTestr pH Meter (Eutech Instrument Pte Ltd., Singapore). The water sample was immediately transferred to the laboratory under controlled temperature conditions. Subsequently, 20 µl of the collected surface water was spread on Luria–Bertani (LB) agar plates. The pH of the culture medium was adjusted to 7.7 to mimic the recorded *in situ* environmental parameters. These plates were incubated at 37 °C for overnight growth, mimicking the SWT measured at the time of collection. An uninoculated blank control plate was also set up in parallel to monitor for any potential contamination introduced during the experimental procedure. Further, a pure bacterial isolate strain named H39_3 was obtained by streak-plate isolation. The colony from the mother plate was re-streaked twice, first to confirm purity and then to obtain the final pure culture for downstream analysis.

### Morphological characteristics

The isolated strain H39_3 was examined under a bright-field microscope (Bx53, Olympus, Japan; 1,000× magnification) to determine the cell shape and Gram staining characteristics. Furthermore, field emission scanning electron microscopy (FESEM) of strain H39_3 was undertaken to confirm the morphological characteristics. Briefly, a fresh culture of strain H39_3 was set up in LB broth, and subsequently, the cells were harvested from an overnight culture by centrifugation at 5,000 r.p.m. The cell pellet was washed using PBS. The washed cell pellet was soaked in a 2.5% glutaraldehyde solution (prepared in PBS) for 8 h at 4 °C. This was followed by subsequent post-fixation with 1% osmium tetroxide. Stepwise dehydration was carried out using ethanol gradients of 10%, 30%, 50%, 70%, 80%, 90% and finally absolute ethanol. Finally, the cells were drop-cast, desiccated and coated with gold:palladium (20:80); FESEM was performed using a Zeiss SUPRA55VP (Carl Zeiss AG, Germany).

### Genomic DNA extraction

A modified version of the existing phenol–chloroform procedure was used to extract genomic DNA (gDNA) from the growing culture [9,10]. Briefly, the cells were pelleted for 5 min at 6,000 r.p.m. A lysis buffer (100 µl of 50 mM Tris-HCl, 100 µl of 20 mM EDTA, 100 µl of 400 mM NaCl, 100 µl of 750 mM sucrose and 10 µl of 10% SDS; Merck, India) was added to the resulting pellet, and it was then incubated for 30 min at 50 °C in a water bath. Proteinase K (10 mg ml^−1^; 5 µl) (HiMedia Laboratories, India) was added, and the mixture was incubated for 3–4 h at 55 °C. Furthermore, 10 µl of lysozyme (10 mg ml^−1^; Merck, India) was added, and the mixture was incubated for 1–2 h at 37 °C. To separate the DNA into the aqueous phase, phenol and chloroform (Merck, India) were added in a 1:1 ratio; the mixture was gently stirred for 5 min, left to stand for 15 min, and then centrifuged for 12 min at 12,000 r.p.m. Subsequently, 60 µl of 3 M sodium acetate (Merck, India) and 750 µl of 100% ethanol (Merck, Germany) were added for overnight incubation; gDNA was precipitated at −20 °C. The centrifugation was undertaken at 12,000 r.p.m. for 12 min to precipitate gDNA and was subsequently dissolved in 20 µl of Tris-HCl (10 mM, pH 8.0). The extracted gDNA was visualized by gel electrophoresis (1% agarose gel) using ethidium bromide (EtBr) fluorescent dye (Merck, India). The NanoDrop 2000c (Thermo Fisher Scientific, United States of America) was used to quantify the yield of gDNA.

### Genotypic identification

Initially, the 16S rRNA amplicon was obtained from the gDNA of strain H39_3 using eubacterial primers (Fc27 and Rc1492; [11]). The PCR reaction consisted of 0.25 µl of DNA DreamTaq polymerase (5 U µl^−1^; Thermo Fisher Scientific, USA), 2.5 µl of 10× DreamTaq buffer, 2.5 µl of 2 mM deoxynucleoside triphosphates (dNTPs), 2 µl of 25 mM magnesium chloride (MgCl_2_), 0.5 µl of each 25 µM primer, 0.25 µl of DNA template, 0.5 µl of BSA (1 mg ml^−1^) and nuclease-free water to make a final volume of 25 µl. The PCR conditions were as follows: initial denaturation at 95 °C for 10 min, 30 cycles of 95 °C for 1 min, 55 °C for 1 min, 72 °C for 3 min and final extension at 72 °C for 20 min. The PCR product was purified using the Qiagen Gel Purification Kit (Qiagen, United States of America) following the manufacturer’s instructions. The purified amplicon was sequenced using Sanger sequencing chemistry with the BigDye Terminator Cycle Sequencing Kit (Thermo Fisher Scientific, United States of America) on an ABI Prism 3500 Genetic Analyzer (Thermo Fisher Scientific, USA). BioEdit (v7.7.1.0) was used to verify the quality of the obtained raw chromatograms. Sequence identification was performed using the BLASTn algorithm against the GenBank/DDBJ/ENA databases (INSDC DNA databases). For constructing the 16S rRNA phylogeny, six near-full-length 16S rRNA sequences representing all species of the genus *Rossellomorea* were downloaded from nucleotide databases (GenBank/ENA/DDBJ), along with 12 additional sequences representative of the Bacillaceae family. These 18 sequences, along with the outgroup *Arthrobacter agilis,* were subsequently aligned with the muscle algorithm [12] in mega version 11 [13]. Before constructing the phylogenetic tree, the best model was selected using jModelTest [14]. Based on the best model with the lowest Bayesian information criterion (BIC) value substitution model (TIM3+I+G), a maximum-likelihood tree was constructed in IQTree [15]. The robustness of the clades in the phylogenetic tree was determined using bootstrap analysis based on 1,000 replicates [16].

For whole-genome sequencing, extracted gDNA was quantified using a Qubit 3.0 fluorometer with the 1× dsDNA HS (High Sensitivity) Assay kit (Thermo Fisher Scientific, United States of America). The Native Barcoding Kit (SQK-NBD114.24, Oxford Nanopore Technologies, United Kingdom) was used to sequence the whole genome. Using long-read sequencing chemistry, the entire genome library was sequenced on the MinION platform (Oxford Nanopore Technologies, United Kingdom). Quality check was performed on the obtained raw reads (Q>8). Adapters were eliminated using Porechop (v0.2.0), and a further quality report of the data was obtained using NanoPlot [17]. Flye (v2.9.3; [18] was used to assemble the *de novo* genome. The programme CheckM [19] was used to measure genome completeness and contamination. QUAST (v5.2.0 [20]) was used to evaluate assembled genome quality. Prokka (v1.14.6 [21]) was used to annotate the draft genome [21]. Proksee was used to generate and visualize the circular map of the entire genome [22].

### Genome data processing

The Type (Strain) Genome Server (TYGS) (https://tygs.dsmz.de) was used to conduct whole-genome sequence-based phylogeny [23]. TYGS uses the Genome blast Distance Phylogeny (GBDP) method to measure genomic distances. Based on inter-genomic distances, the evolutionary tree was inferred with branch support from 100 replicates using FASTME 2.1.4 [24]. The branch lengths were scaled in terms of the GBDP distance formula d5. The tree was rooted at the midpoint.

MiGA was used to perform taxonomic classification, essential gene presence analysis and average amino acid identity (AAI) (https://disc-genomics.uibk.ac.at/miga [25]). AntiSMASH was used to identify biosynthetic gene clusters (BGCs) that encode natural products, also known as secondary metabolites [26]. The average nucleotide identity (ANI) between genomes was assessed using the EZBioCloud ANI calculator (Chalita *et al*., 2024)[27], which implements the Orthologous Average Nucleotide Identity (OrthoANIu) algorithm [28] and the Fast alignment-free computation of whole-genome average nucleotide identity (FastANI) algorithm, an alignment-free MinHash-based method [29]. Digital DNA–DNA hybridization (dDDH) values were calculated using the Genome Genome Distance Calculator (GGDC) [30]. Antimicrobial resistance genes were identified and annotated using the resistance gene identifier (RGI) module of the Comprehensive Antibiotic Resistance Database (CARD) [31].

The GhostKOALA-KEGG Mapper was used to infer functional pathways [32,33]. Carbohydrate active enzymes (CAZymes) were annotated using the dbCAN3 server [34]. *In silico* phenotyping was performed using Traitar (https://github.com/hzi-bifo/traitar) [35].

## Results

**Fig. 1.**
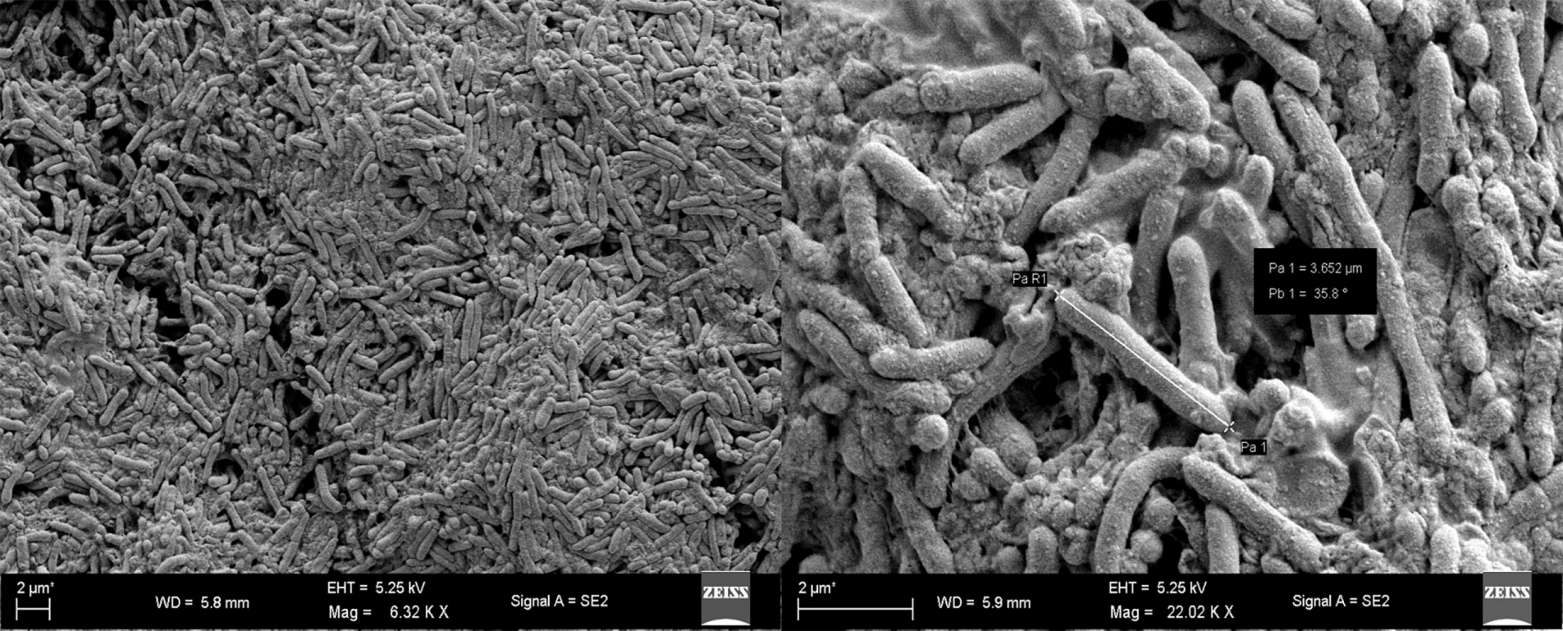
FESEM image of *Rossellomorea* sp*.* strain H39_3. A scale bar is shown at the bottom left of the image. Mag: magnification, EHT: Electron High Tension, WD: working distance.

**Fig. 2.**
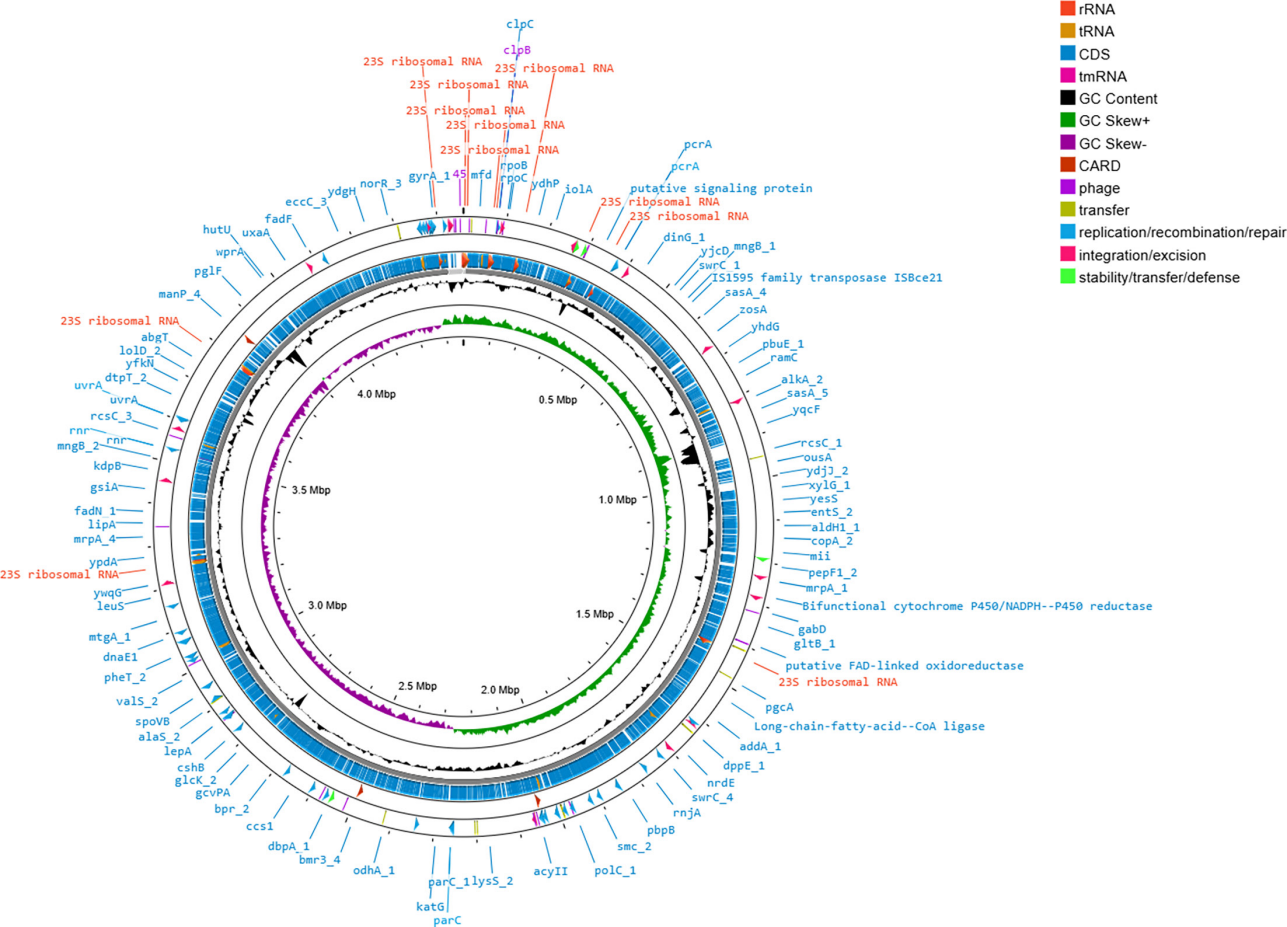
Map showing a draft genome circular map of the strain H39_3, assembled into three contigs (representation only); markings on the outermost ring show identified genes based on Prokka annotation, including CDSs, rRNA, tRNA and tmRNA. The middle ring illustrates G+C content as deviations relative to the genome average. The innermost ring shows G+C skew, with positive values highlighted in green and negative values in purple. tmRNA: transfer-messenger RNA.

**Fig. 3.**
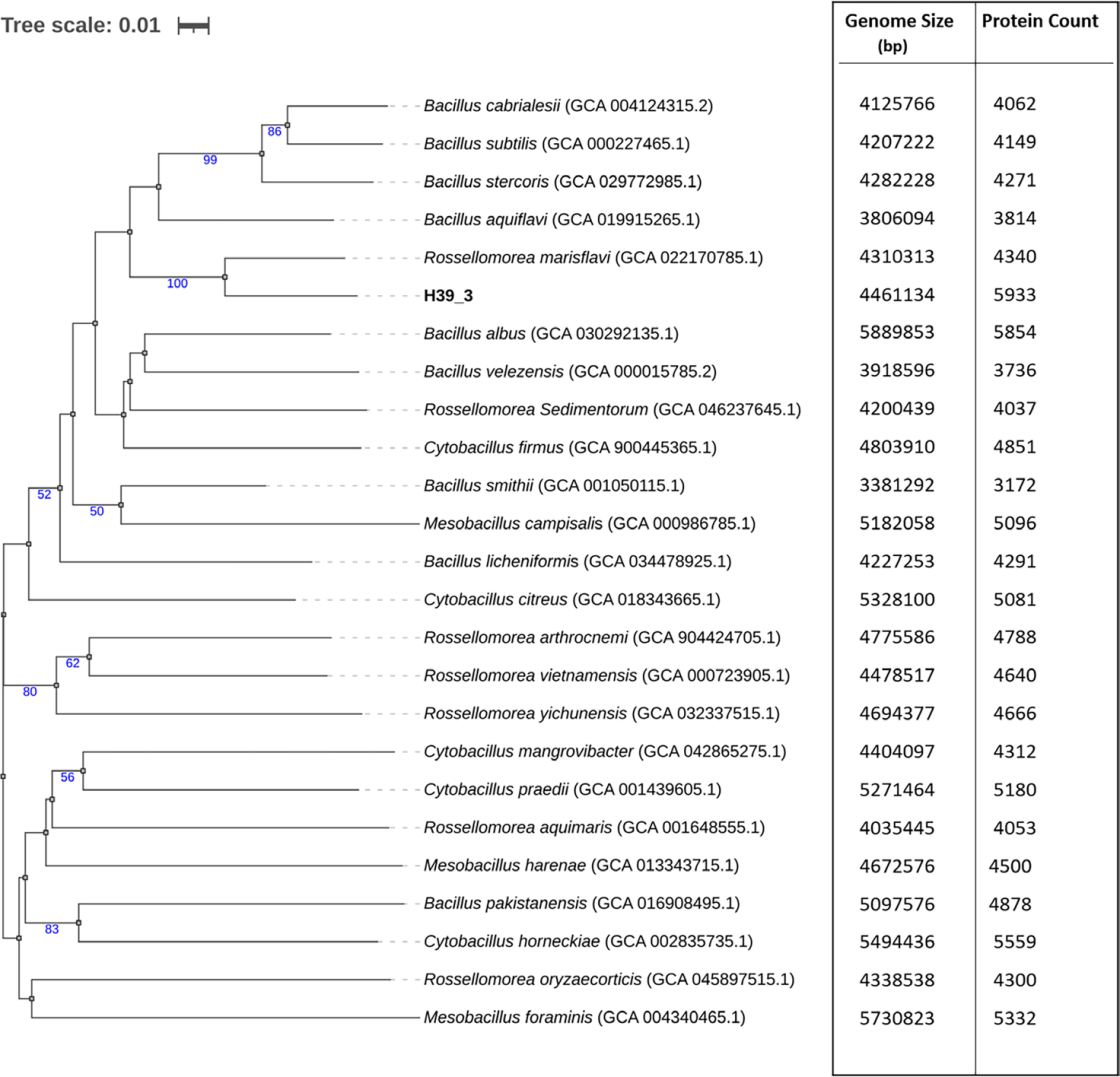
Phylogram showing the phylogenetic relationship of strain *Rossellomorea* sp. H39_3 with other known species of *Rossellomorea* and selected representative within the family Bacillaceae. The phylogeny was inferred using the GBDP method implemented on the TYGS server. Distances were calculated from whole-genome sequences, and the tree was constructed using FastME v2.1.6.1. Branch lengths are scaled in terms of the GBDP distance formula d5, indicating genome-wide dissimilarity. Numbers (in blue) at nodes represent GBDP pseudo-bootstrap support values >50%, derived from 100 replications. The adjacent table on the right-hand side shows the size of genome and protein counts of the respective strains presented in the tree.

The bright-field microscopy revealed that strain H39_3 is a Gram-positive, rod-shaped bacterium, and it was further verified based on FESEM (Fig. 1). The sequenced 16S rRNA data of strain H39_3 shared the highest identity with *Rosssellomorea marisflavi* (query cover: 100%; percent identity: 100%) on the basis of blastn analysis, supporting the taxonomic affiliation of strain H39_3 to the genus *Rossellomorea*. In the 16S rRNA-based phylogenetic tree, strain H39_3 formed a distinct monophyletic clade with *Rosellomorea marisflavi* (Fig. S1, available in the online Supplementary Material). The assembled draft genome of strain H39_3 consisted of three contigs, and the size was 4,461,134 bp (Fig. 2). The G+C content of the genome was 48.64 mol%. The identified closest relative of strain H39_3 was *R. marisflav*i. The dDDH, orthoANI and AAI values of strain H39_3 exhibited the highest values of 46.1%, 92.23% and 98.7%, respectively, with *R. marisflav*i. The whole-genome-based phylogeny also confirmed the closest affiliation with *R. marisflav*i. The basic genome features and associated quality statistics are detailed in Table 1. The genome completeness was ~95%, and contamination was 0.75%, reflecting an acceptable benchmark for a high-quality draft genome [36]. The predicted number of genes present in the genome was 6,061, with 5,913 CDSs (Fig. S2, available in the online Supplementary Material). The dDDH computed by GGDC between the whole genome of strain H39_3 and its closest relative was 46.1%, which is less than the accepted cutoff value of 70% for species delineation (Table 2). Additionally, ANI value of the strain with *R. marisflav*i was found to be 92.23% based on OrthoANI and 92.41% based on FastANI. Both the values were significantly below the acceptable threshold for ANI (95–96%) [28,37]. These results strongly suggest that strain H39_3 is a potential novel species, as per proposed standards for species delineation based on whole-genome-based relatedness metrics [37,39]. Despite this, 16S rRNA showed 100% identity with *R. marisflavi*. This highlights that although 16S rRNA is a valuable marker, it can fail to capture genome-wide divergence and may be insufficient as a sole criterion for species-level identification in this genus [40,42]. However, formal species description is awaited, following the completion of robust chemotaxonomic and phenotypic characterizations. The value of AAI was 98.7% with *R. marisflav*i, reflecting conserved protein-coding genes, though the lower ANI (≈92%) signals broader genomic divergence in noncoding regions. Based on the GBDP tree, the isolate formed a separate monophyletic clade with *Rosellomorea marisflavi*. The tree branch was well supported by a bootstrap value of 100 (Fig. 3).

**Table 1.** Comparison of the genome of *Rossellomorea* sp. strain H39_3 with genomes of all reported species representing this genus

Reference genome	DDH (%)	ANI (%)	AAI (%)	GGDC
*R. marisflavi*	46.1	92.23	98.7	0.0814
*R. oryzaecorticis*	21	72.47		0.2092
*R. arthrocnemi*	19.7	72.6		0.2233
*R. aquimaris*	19.4	71.59	80.2	0.226
*R. vietnamensis*	19.3	73.14	79.9	0.2277
*R. pakistanensis*	21.2	69.22		0.2068

**Table 2.** Draft genome statistics of *Rossellomorea* sp. strain H39_3

Genome feature	No. or length
Size	4,461,134 bp
No. of contigs	3
No. of large contigs (>1,000 bp)	3
Contig N50	4,414,898
Contig N90	4,414,898
L50	1
L90	1
G+C (mol%)	48.64

The genome annotation of strain H39_3 revealed the presence of motility-related genes. For example, genes encoding flagellar motor-mounted switch complex proteins (fliG, fliN, fliM, fliY), flagellar basal body proteins (flgCEFG), hook-basal body (fliE) and flagellar biosynthesis proteins and regulators (fliRO and flhF) were identified from the draft genome. The CAZymes involved in complex carbohydrate degradation, including glycoside hydrolases (GHs), polysaccharide lyases (PLs), carbohydrate esterases (CEs) and synthesis, were present in the genome [43]. The studied genome possesses genes from 15 different families of GHs, six different families of glycosyltransferase and six different families of CEs. Based on dbCAN analysis, there is a notably higher occurrence of CAZyme families predicted to act on pectin, including pectate lyase enzyme from the PL9 family, CE8 (pectin methylesterase), CE912 (pectin acetyltransferase), GH28 (polygalacturonases) and GH105 (unsaturated glucuronyl/galacturonylhydrolase). The genome has the highest number of hits (68) corresponding to GH13 family enzymes, which are major α-amylases mainly involved in catalysing the hydrolysis of polysaccharides such as glycogen and starch [8,44]. The substrate utilization annotation with respect to CAZymes gene families revealed the *in silico* ability of this bacterium to break down chitin, pectin, hemicellulose, inulin, sucrose and starch, among others. *In silico* phenotyping by Traitar indicated that the organism is motile and Gram-negative. The isolate produces enzymes including oxidase, catalase and lipase and is susceptible to bile. It can convert nitrate to nitrite and nitrite to gas. The isolate can use tartrate, trehalose, cellobiose, acetate and mucate. The isolate can grow on ordinary blood agar, MacConkey agar and in the presence of high NaCl (6.5%) concentration, based on *in silico* phenotyping. The results of *in silico* phenotyping are summarized in Fig. S3 (available in the online Supplementary Material). The *in silico* analysis predicted a Gram-negative phenotype; however, microscopic observations confirmed that the strain is Gram-positive. Such mismatches can happen, as the accuracy of *in silico* phenotype predictions is affected by factors like genome completeness and can be less reliable for certain or less-studied, environmentally diverse taxa [35]. Thus, these observed features need to be validated by robust experimental approaches.

The BGCs identified by AntiSMASH included Thiopeptide-Linear azole (in)e-containing peptides (LAP), Lanthipeptide-Class IV, Terpene and Type III polyketide synthases (T3PKS). The antibiotic resistome of the isolate contains vanW, vanY and qacG genes, as predicted using the RGI database (strict hits only). The genome also showed the presence of the arsC (arsenate reductase) gene, encoding the ArsC protein, indicating the potential ability to reduce arsenate to arsenite. Additionally, a gene encoding the arsenical pump membrane protein YdfA was identified, suggesting the capability of the strain to mobilize or export arsenite from the cytoplasm. The genome also encodes numerous genes, including cysN, cysC and sqr, which are known to be involved in sulphur metabolism.

The functional pathways inferred by GhostKOALA-KEGG Mapper showed that *Rossellomorea* sp. strain H39_3 contains the nitrate assimilation gene nirA, linked to nitrogen metabolism.

Overall, this newly isolated bacterium strain encompasses a suite of functional traits indicative of multifaceted ecological significance. In particular, the presence of an array of genes linked to the metabolism of carbon, nitrogen and sulphur reflects the genomic potential for application towards the bioremediation of contaminated flowing aquatic ecosystems, such as the River Hindon. The genome-based insights are valuable for establishing a baseline for functional validation and to conduct further studies aimed at practical applications in the field of bioremediation.

## Supplementary material

10.1099/acmi.0.001042.v4 Uncited Supplementary Material 1.

## Abbreviations

AAI: average amino acid identity
ANI: average nucleotide identity
BGCs: biosynthetic gene clusters
CARD: Comprehensive Antibiotic Resistance Database
CAZymes: carbohydrate active enzymes
CEs: carbohydrate esterases
dDDH: digital DNA–DNA hybridization
FastANI: fast alignment-free computation of whole-genome average nucleotide identity
FESEM: field emission scanning electron microscopy
GBDP: Genome BLAST Distance Phylogeny
gDNA: genomic DNA
GGDC: Genome Genome Distance Calculator
GHs: glycoside hydrolases
INSDC: International Nucleotide Sequence Database Collaboration
LB: Luria–Bertani
PLs: polysaccharide lyases
RGI: resistance gene identifier
SWT: surface water temperature
TYGS: Type (Strain) Genome Server
