# Supplementary material for: Genome description of a potentially novel species of Rossellomorea sp. strain H39_3 isolated from the Hindon River, India
Source: Access Microbiol. 2026 May 19;8(5):001042.v4. doi: 10.1099/acmi.0.001042.v4 (PMC13186505; doi:10.1099/acmi.0.001042.v4)

Supplementary data

Genome description of a potentially novel species of *Rossellomorea* sp. strain H39_3 isolated from Hindon River, India

Chakresh Kumar[1], Nirupama Saini[1], Anwesha Ghosh[2] and Punyasloke Bhadury[1, 2, *]

[1]Integrative Taxonomy and Microbial Ecology Research Group, Department of Biological Sciences, Indian Institute of Science Education and Research Kolkata, Mohanpur-741246, Nadia, West Bengal, India

[2]Centre for Climate and Environmental Studies, Indian Institute of Science Education and Research Kolkata, Mohanpur-741246, Nadia, West Bengal, India

*Corresponding author: pbhadury@iiserkol.ac.in

Supplementary Figures

Fig. S1: Maximum likelihood tree based on 16S rRNA sequences showing the phylogenetic position of strain H39_3. The tree was generated using IQTree and is based on TIM3+I+G model. The numbers above branches are bootstrap support values >50% from 1000 replications. NCBI accession numbers are mentioned in parentheses. Arthrobacter agilis was used as an outgroup.

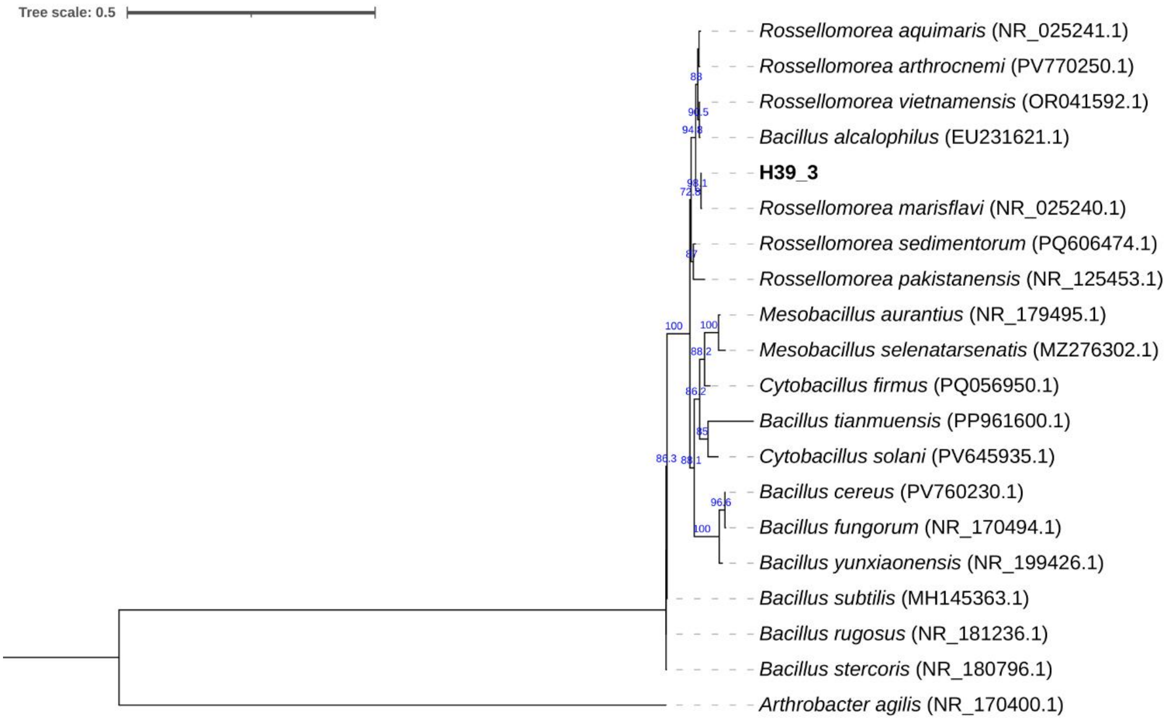

Fig.S2: Genome map of *Rossellomorea* sp. H39_3 in comparison with all the type strains of genus *Rossellomorea*. The circular genome map also shows the GC content and GC skew (+/-).

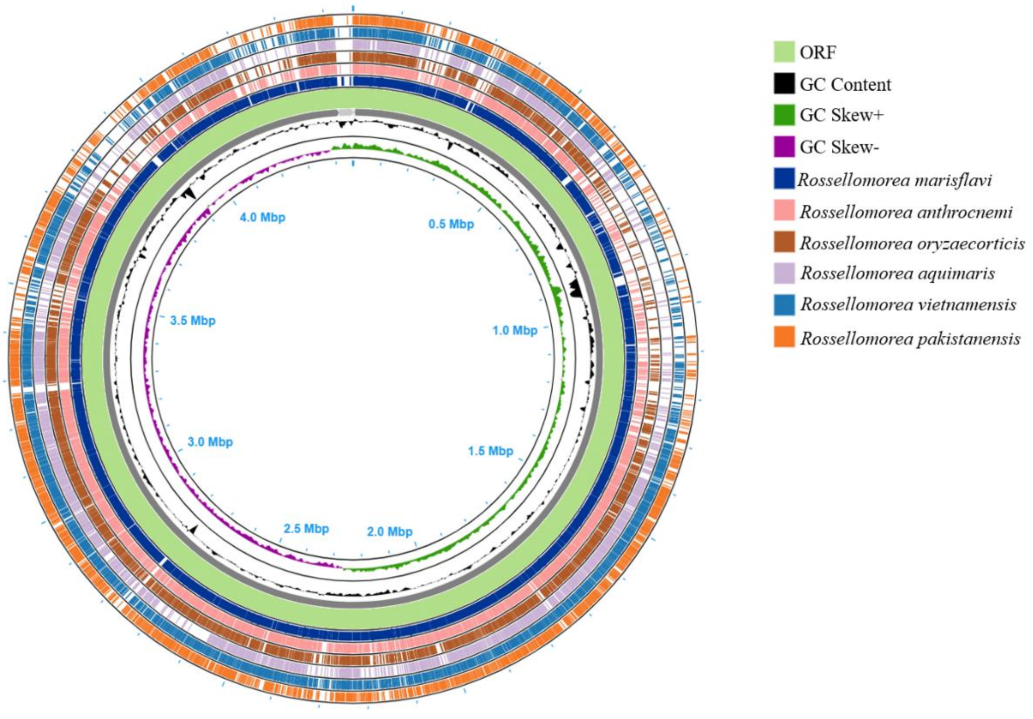

.

Fig. S3: *In silico* phenotypic data showing functional traits as deduced from genome sequence data of strain H39_3 using Traitar.

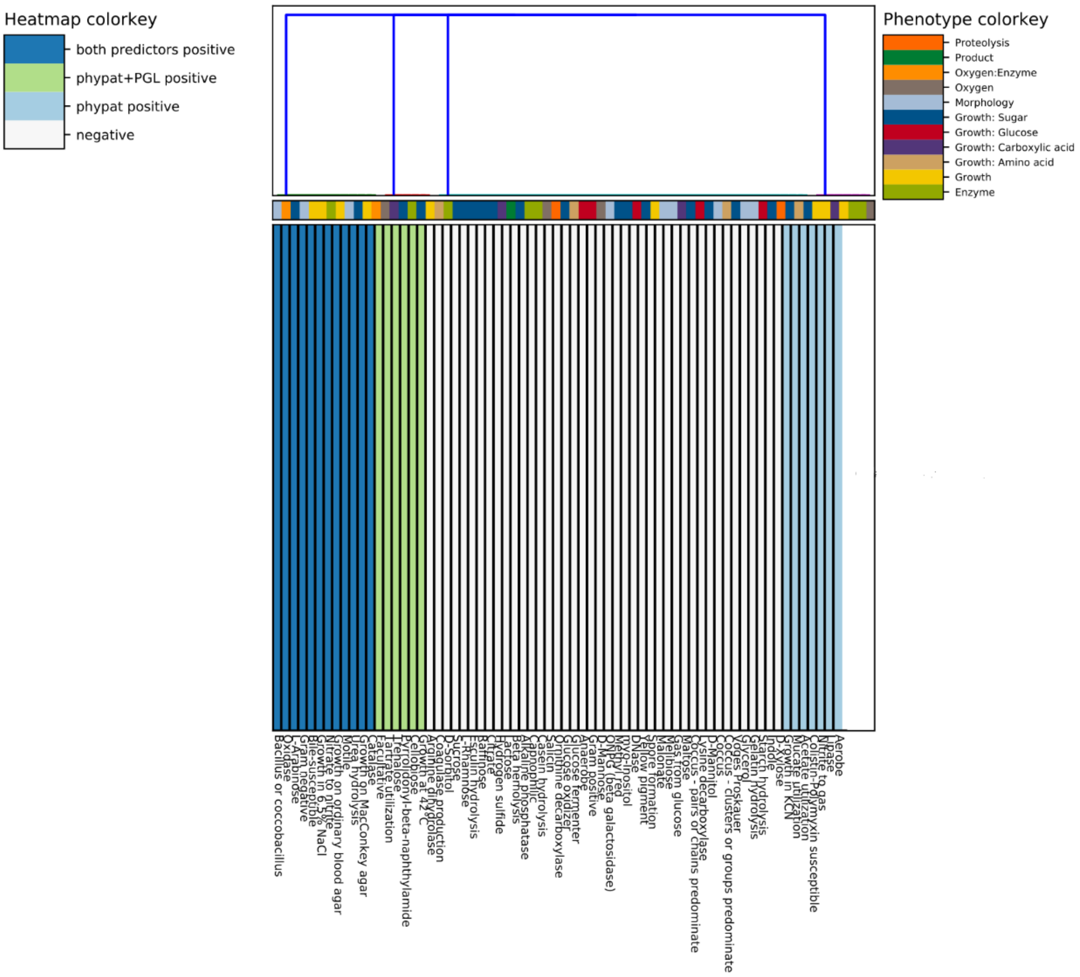

Supplement: Uncited Supplementary Material 1. [file acmi-8-01042-s001.pdf]
